# Internal reference updating in visual duration discrimination: A search for boundary conditions: Internal reference updating

**DOI:** 10.3758/s13414-026-03235-6

**Published:** 2026-04-10

**Authors:** Paul Kelber, Rolf Ulrich, Karin Maria Bausenhart, Roman Liepelt, Ruben Ellinghaus

**Affiliations:** 1 Faculty of Science, Department of Psychology, Cognition and Perception, University of Tübingen, Schleichstraße 4, 72076 Tübingen, Germany; 2 Faculty of Psychology, Department of General Psychology, Judgment, Decision Making, Action, FernUniversität in Hagen, Hagen, Germany

**Keywords:** Comparative judgment, Duration discrimination, Time order error, Type B effect, Sensation weighting, Internal reference

## Abstract

A growing body of psychophysical research suggests that the discrimination of successive stimuli involves more than taking the difference between their sensation magnitudes, challenging traditional difference models. Two elaborated theories assume that each sensation magnitude is weighted by its reference level (sensation weighting model) or that the second sensation magnitude is compared to a dynamically updated internal reference, in which the first sensation magnitude gravitates towards the previous reference level (internal reference model). Whereas both models can explain higher discrimination sensitivity when the standard precedes the comparison (negative Type B effect), only the sensation weighting model can account for higher discrimination sensitivity when the standard follows the comparison (positive Type B effect). Most previous studies reported negative Type B effects, while some positive Type B effects have also been reported, especially for duration discrimination with short stimulus durations and/or short inter-stimulus intervals combined with adaptive-staircase procedures. The present study systematically searched for positive Type B effects in visual duration discrimination by orthogonally varying stimulus duration (80 vs. 500 ms standard), inter-stimulus interval (200 vs. 900 ms), and stimulus type (filled vs. empty intervals) across four experiments using the method of constant stimuli. Type B effects were consistently negative across all experimental conditions and analysis methods. The absence of positive Type B effects is consistent with the internal reference model and casts doubt on whether the additional flexibility in the sensation weighting model is needed to explain visual duration discrimination sensitivity.

## Introduction

How do we discriminate the magnitudes of our sensations? This fundamental question is routinely investigated by presenting two stimuli in succession and asking observers which one appears larger along a given physical dimension. For example, in duration discrimination, observers typically have to judge which of two temporal intervals lasted longer.^1^ Typically, each trial involves the discrimination between a standard *s*, which is constant across trials (e.g., 500 ms), and a comparison *c*, which varies across trials (e.g., 300–700 ms). This results in a psychometric function *F* that represents the probability of judging *c* to be longer than *s* as a function of *c* and is usually S-shaped. Most saliently, *F* is formed by (1) the constant error (difference between the point of subjective equality, PSE, and *s*), which determines its horizontal location, and (2) the variable error (difference limen, DL), which determines its slope. First, the PSE reflects the midpoint of *F*, at which *s* and *c* are equally likely to be judged as longer (i.e., $\text {PSE} = c_{.50}$). The more the PSE deviates from *s*, the more biased the stimulus discrimination. Second, the DL reflects the spread and shallowness of *F*, often defined as half the interquartile range of *F* (i.e., $\text {DL} = (c_{.75} - c_{.25}) \, / \, 2$). The flatter *F* becomes, the higher the DL and thus the lower the discrimination sensitivity. As described next, several theories of comparative judgment make different predictions about whether and how discrimination sensitivity depends on the presentation order of *s* and *c*.

A plausible starting point is to assume that in each trial, *s* and *c* are discriminated by comparing their perceived magnitudes (e.g., durations). This is assumed by Thurstonian difference models (Thurstone, 1927a, b), such as standard models for two-alternative forced-choice (2AFC) tasks in signal detection theory (see Green & Swets, 1966, pp. 64–69; Macmillan, 2004, pp. 165–179; Wickens, 2002, pp. 96–103) and related models (e.g., Luce, 1963, Yeshurun et al., 2008). Formally, these models posit that observers take the difference between the perceived durations of the stimuli presented first and second, $\textbf{D} = \textbf{X}_1 - \textbf{X}_2$, and compare this difference against a criterion $\gamma$. The first (second) stimulus is judged to be longer if $\textbf{D} > \gamma$ ($\textbf{D} \le \gamma$), where $\gamma = 0$ ms reflects unbiased discrimination. In each trial, *s* may precede *c* (order $\langle sc \rangle$) or follow *c* (order $\langle cs \rangle$). As shown by Dyjas et al. (2012, p. 1835), difference models predict that the psychometric functions for these two stimulus orders, $F_{\langle sc \rangle }$ and $F_{\langle cs \rangle }$, differ in location by $2\gamma$, but are identical in shape. Accordingly, the PSE may vary between $\langle sc \rangle$ and $\langle cs \rangle$, whereas the DL should be unaffected by stimulus order.

However, empirical evidence suggests that stimulus order in discrimination tasks modulates not only the PSE but also the DL. Effects of stimulus order on the PSE (e.g., Fechner, 1860; Guilford, 1954; Hellström, 1979; Stott, 1935; Woodrow, 1935), termed *Type A effects* (Ulrich & Vorberg, 2009), can be accounted for by difference models as response biases ($\gamma \ne 0$ ms). But crucially, the DL has also often been found to be sensitive to stimulus order (e.g., Ellinghaus et al., 2024; Martin & Müller, 1899). These so-called *Type B effects* (Ulrich & Vorberg, 2009) contradict the aforementioned prediction of traditional difference models.^2^ By convention, the Type B effect is calculated as $\text {DL}_{\langle sc \rangle } - \text {DL}_{\langle cs \rangle }$. And by implication, a positive Type B effect reflects $\text {DL}_{\langle sc \rangle } > \text {DL}_{\langle cs \rangle }$, that is, a higher discrimination sensitivity for the order $\langle cs \rangle$. Conversely, a negative Type B effect reflects $\text {DL}_{\langle sc \rangle } < \text {DL}_{\langle cs \rangle }$, that is, a higher discrimination sensitivity for the order $\langle sc \rangle$. Unlike the traditional difference models, two elaborated theories of comparative judgment (e.g., duration discrimination) can explain Type B effects: the *internal reference model* (Dyjas et al., 2012; Lapid et al., 2008; for similar accounts, see Michels & Helson, 1954; Nachmias, 2006) and the *sensation weighting model* (Hellström, 1979, 1985, 2003).

The internal reference model assumes that discriminating between two successive stimuli involves comparing the perceived magnitude of the later stimulus with a memory representation of the former stimulus magnitude. In this memory representation, the perceived magnitude of the former stimulus is shifted toward recent sensation magnitudes through a dynamic updating process. Specifically, observers discriminate the durations of two stimuli presented successively in trial *n* by comparing the perceived duration of the stimulus presented second in this trial, $\textbf{X}_{2,\,n}$, against an internal reference, $\textbf{I}_n$:

1 $$\begin{aligned} \textbf{D}_n = \textbf{I}_n - \textbf{X}_{2,\,n}. \end{aligned}$$

The internal reference $\textbf{I}_n$ reflects a weighted sum of the perceived duration of the stimulus presented first in this trial, $\textbf{X}_{1,\,n}$, and the internal reference from the previous trial, $\textbf{I}_{n-1}$, and it is dynamically updated according to

2 $$\begin{aligned} \textbf{I}_n = g \cdot \textbf{I}_{n-1} + (1-g) \cdot \textbf{X}_{1,\,n}, \end{aligned}$$

where the weight $g \in [0, 1)$ describes the relative contribution of the stimulus history. In each trial *n*, the first (second) stimulus is judged to be longer if $\textbf{D}_n > \gamma$ ($\textbf{D}_n \le \gamma$).^3^ The core idea of the internal reference model—assimilation of previous and current information—also underlies several Bayesian observer models (see, e.g., de Jong et al., 2021; Glasauer & Shi, 2021; Jazayeri & Shadlen, 2010; Raviv et al., 2012; Shi et al., 2013; Wiener et al., 2014;).

It has been shown that the internal reference model predicts negative Type B effects if $g > 0$ irrespective of whether stimulus order is varied across blocks (Dyjas et al., 2012, pp. 1837–1840) or across trials (Dyjas & Ulrich, 2014, pp. 1145–1148). Intuitively, this prediction arises because the information-rich perceived duration of *c* enters into $\textbf{D}$ with full weight for $\langle sc \rangle$, but not for $\langle cs \rangle$, where it becomes more blurred by previous sensations the larger *g* is. Only in the extreme case of $g = 0$ does the internal reference model reduce to a difference model and produce a null Type B effect. Importantly, however, the internal reference model cannot produce positive Type B effects.

This contrasts with Hellström’s established sensation weighting model, which explains Type A effects as perceptual phenomena (Hellström, 1977, 1978, 1979, 1985), and flexibly accounts for negative, null, and positive Type B effects (Hellström, 2003; Hellström et al., 2020; Hellström & Rammsayer, 2004, 2015). Unlike the internal reference model, the sensation weighting model proceeds from the idea that not only the first but also the second effective magnitude is retrieved from memory. Building on adaptation level theory (Helson, 1947, 1964), the effective magnitudes of the first and the second stimulus are described as separate weighted sums of the respective sensation magnitude (perceived durations $\textbf{X}_1$ and $\textbf{X}_2$) with its reference level ($\textbf{R}_1$ and $\textbf{R}_2$). Thus,

3 $$\begin{aligned} \textbf{D} = [s_1 \textbf{X}_1 + (1 - s_1) \textbf{R}_1] - [s_2 \textbf{X}_2 + (1 - s_2) \textbf{R}_2], \end{aligned}$$

where each sensation magnitude is drawn towards (away from) its reference level if its weight *s* is below (above) 1, thus capturing assimilative (contrastive) anchoring effects. Again, the first (second) stimulus is judged to be longer if $\textbf{D} > \gamma$ ($\textbf{D} \le \gamma$). Hellström’s model attributes Type B effects to the differential weighting of the two sensation magnitudes: Stronger weighting of the second stimulus ($s_1 < s_2$) produces a negative Type B effect, equal weighting ($s_1 = s_2$) a null Type B effect, and stronger weighting of the first stimulus ($s_1 > s_2$) a positive Type B effect (e.g., Hellström & Rammsayer, 2004).

Reliable observations of positive Type B effects would provide critical evidence for the sensation weighting model and against the internal reference model. However, many previous studies found negative Type B effects for the discrimination of various sensation magnitudes, such as weight (Martin & Müller, 1899; Ross, 1964), visual height (von Castell et al., 2017), visual and auditory numerosity (Ellinghaus et al., 2018; Lapid et al., 2009), visual and auditory intensity (Ellinghaus et al., 2021, 2018), as well as visual and auditory frequency (Ellinghaus et al., 2018; Nachmias, 2006). Furthermore, as reviewed by Ellinghaus et al. (2024), most studies on duration discrimination found negative Type B effects (e.g., Ellinghaus et al., 2019; Ellinghaus et al., 2021; Gao et al., 2021; Gordon, 1967; Grondin and McAuley, 2009; Lapid et al., 2008; Thönes et al., 2018; Ulrich et al., 2006; van Allen et al., 1966). But importantly, Hellström and Rammsayer (2004, 2015) and Hellström et al. (2020) also observed positive Type B effects—along with many negative Type B effects—in some conditions (short stimulus durations and/or short ISI) of their extensive duration discrimination experiments (see Table 1). These findings challenge the internal reference model. Taken together, Ellinghaus et al. (2024) found meta-analytic evidence for a negative Type B effect in the discrimination of magnitudes other than duration and in duration discrimination with relatively long stimulus durations ($s \ge 500$ ms), but inconclusive evidence in duration discrimination with shorter durations.

**Table 1:** Overview of experimental conditions yielding positive Type B effects in previous duration discrimination studies

Study and experiment	Modality	Stimulus type	Standard duration *s*	ISI	Procedure
Hellström and Rammsayer (2004, Experiment 1)	Auditory	Filled	50 ms	100 ms	Adaptive
Hellström and Rammsayer (2004, Experiment 1)	Auditory	Filled	50 ms	300 ms	Adaptive
Hellström and Rammsayer (2015, Experiment 1)	Auditory	Filled	100 ms	900 ms	Adaptive
Hellström and Rammsayer (2015, Experiment 1)	Visual	Empty	100 ms	900 ms	Adaptive
Hellström and Rammsayer (2015, Experiment 2)	Auditory	Filled	215 ms	900 ms	Adaptive
Hellström and Rammsayer (2015, Experiment 2)	Auditory	Filled	464 ms	900 ms	Adaptive
Hellström et al. (2020, Experiment 3)	Visual	Empty	100 ms	900 ms	Adaptive

Why did most studies observe negative Type B effects? According to Hellström et al. (2020, p. 3212), “this fact seems to be due to researchers’ strange reluctance to use interstimulus intervals other than about 1000 ms, or stimuli briefer than 500 ms.” Indeed, several experiments with shorter stimulus durations and/or ISIs revealed positive Type B effects in both auditory and visual duration discrimination (see Table 1). This pattern is consistent with Hellström’s hypothesis (e.g., Hellström, 1979, p. 171) that shorter stimulus durations and ISIs lead to forward masking of the second stimulus, effectively leading to a stronger weighting of the first stimulus and thus to a positive Type B effect, whereas longer stimulus durations and ISIs lead to information loss about the first stimulus, effectively leading to a stronger weighting of the second stimulus and thus to a negative Type B effect. However, when examining duration discrimination sensitivity for filled auditory intervals with different combinations of shorter and longer stimulus durations and ISIs, Bausenhart et al. (2015) mostly found negative Type B effects, which only vanished (but did not reverse) when employing both short stimulus durations ($s = 100$ ms) and a short ISI (300 ms). This finding may be explained by assuming that dynamic reference-level integration is under cognitive control (Dyjas et al., 2014) and therefore possibly too slow to influence the comparison mechanism when stimuli are presented in rapid succession (see Bausenhart et al., 2015).

Another striking regularity in Table 1 is that all positive Type B effects were obtained when the DL was measured with an adaptive-staircase procedure rather than with the method of constant stimuli used in many comparable studies and in particular by Bausenhart et al. (2015). This imbalance is worrisome because it is difficult to isolate Type B effects from Type A effects when pairing extremely short stimulus durations with an adaptive-staircase procedure and estimating the DL directly from the *c* values in the staircases without constructing a psychometric function (cf. Sternberg et al., 1982). Specifically, all studies in Table 1 used the weighted up-down method (Kaernbach, 1991) to target the points of the psychometric function at which *c* is judged to be longer than *s* with a probability of 25% and 75% (i.e., $c_{.25}$ and $c_{.75}$). The *c* values at the reversals or all *c* values in the last 20 trials of each staircase were then averaged to arrive at $\hat{c}_{.25}$ and $\hat{c}_{.75}$, and the DL was estimated via $(\hat{c}_{.75} - \hat{c}_{.25}) / 2$. If *s* is extremely short (e.g., 50 ms), the lower run may approach the physical limit of 0 ms without reaching $c_{.25}$ and thus yield an invalid estimate $\hat{c}_{.25}$. In such cases, the DL estimates are shaped not only by slope differences but also by horizontal shifts between the underlying psychometric functions for $\langle sc \rangle$ and $\langle cs \rangle$. This source of potential bias in the Type B effect estimates may further interact with the presentation of lower runs (starting with $c < s$) and upper runs (starting with $c > s$) in separate blocks (Hellström et al., 2020; Hellström & Rammsayer, 2004, 2015) and with the prohibition of *c* to cross *s* in each staircase (as implemented by Hellström and Rammsayer, 2004, 2015, but not by Hellström et al., 2020).

Indeed, exploratory simulations indicate that the internal reference model can produce biased positive Type B effect estimates under the potentially problematic conditions described above. This suggests that previous reports of positive Type B effects may—but need not—reflect response biases rather than differences in discrimination sensitivity, which makes it difficult to distinguish between the internal reference model and the sensation weighting model based on the available data. A systematic search for positive Type B effects with the method of constant stimuli thus seems much needed to advance the theoretical debate. This applies in particular to visual duration discrimination, which yielded arguably the most striking demonstration of a positive Type B effect so far (Hellström et al., 2020, Experiment 3), but did not yet receive any systematic investigation with the method of constant stimuli, akin to the study by Bausenhart et al. (2015) for auditory duration discrimination. Such an investigation should cover filled and empty intervals, as both stimulus types have sometimes (but not consistently) led to positive Type B effect estimates in previous studies (see Table 1).

The present study provides a systematic analysis of Type B effects in visual duration discrimination by orthogonally varying stimulus duration ($s = 80$ vs. 500 ms), ISI (200 vs. 900 ms), and stimulus type (filled vs. empty intervals) across four experiments using the method of constant stimuli. The internal reference model predicts negative-to-null Type B effects in all conditions. Although negative Type B effects can be expected to weaken or even disappear with short stimulus durations and/or ISI due to a shortened integration period, they must not reverse into positive Type B effects if the internal reference model is to be maintained. By contrast, from a formal perspective, “anything goes” in the sensation weighting model. Nonetheless, Hellström’s theoretical considerations and model fits (Hellström, 1979, 1985; Hellström et al., 2020; Hellström and Rammsayer, 2004, 2015; but see also Hellström, 2003) suggest that with short stimulus durations and/or ISI, the first stimulus tends to be weighted more strongly than the second, which would imply a positive Type B effect in the sensation weighting model. Our design choices (short stimulus durations and ISI) thus create conditions most likely to produce positive Type B effects in the sensation weighting model, enabling a critical test of the internal reference model.

## Experiments

Four experiments evaluated the effect of stimulus order on duration discrimination sensitivity (Type B effect) for short versus long durations of filled (Experiments 1 and 3) versus empty visual intervals (Experiments 2 and 4) separated by a short (Experiments 1 and 2) versus long ISI (Experiments 3 and 4). Following Hellström and Rammsayer (2004, 2015), Experiments 1–4 used trial-wise accuracy feedback to improve the capture of the psychometric functions compared to a pilot experiment without accuracy feedback (see Appendix A). This pilot experiment was otherwise similar to Experiment 1 and led to the same conclusion as Experiments 1–4. In line with typical values from previous studies, the long visual stimulus durations were $s = 500$ ms and $c =$ 300–700 ms (e.g., Dyjas et al., 2012) and the long ISI was 900 ms (e.g., Hellström et al., 2020; Hellström & Rammsayer, 2004, 2015). The short visual stimulus durations of $s = 80$ ms and $c =$ 20–140 ms were chosen to be as short as possible without causing appreciable brightness differences between filled intervals *s* and *c* due to Bloch’s law. Specifically, four pilot observers gave absolute ratings (0–10) of the brightness of equiluminant filled intervals ranging in duration from 5 to 100 ms in increments of 5 ms, revealing that brightness ratings rose sharply from 5 to 20 ms but then leveled off. Moreover, the short ISI of 200 ms was determined to be the shortest possible ISI that still produced a distinct perception of two separate intervals rather than indistinct flickering.

### Method

#### Observers

In each experiment, 40 observers completed a single 1-h session (Experiment 1: 30 female, 37 right-handed, mean age [age range]: 23 [19–50] years; Experiment 2: 31 female, 35 right-handed, 25 [18–41] years; Experiment 3: 26 female, 39 right-handed, 25 [19–55] years; Experiment 4: 30 female, 38 right-handed, 25 [19–37] years). Of the total number of 160 observers, 18 replaced original observers that were deemed ineligible due to flat psychometric functions across conditions (one, five, seven, and five replacements in Experiments 1, 2, 3, and 4, respectively). Re-analyses with the original samples (both with and without the additionally recruited observers) led to virtually identical results and the same conclusion about the direction of the Type B effect. The sample size of 40 observers per experiment ensured a statistical power $1-\beta$ of above 95% to detect the negative Type B effect of size $\eta _{p}^{2}=.08$ observed with short stimulus durations in the pilot experiment (calculated with G*Power 3.1.9.7; Faul et al., 2007). All observers reported normal or corrected-to-normal vision and were reimbursed with course credit or 12 €.

#### Apparatus and stimuli

A red light-emitting diode (LED) was lit (filled interval; 77.5 cd/m$^2$) or unlit (empty interval; $< 0.1$ cd/m$^2$), controlled by an Arduino Uno microcontroller and custom Python scripts. Observers viewed the LED from a chinrest at a distance of about 85 cm in a dimly lit chamber. The duration of *s* (80 or 500 ms) was symmetrically surrounded by nine levels of *c* (20–140 ms in steps of 15 ms, or 300–700 ms in steps of 50 ms). These stimulus durations were presented with an accuracy and precision of below 0.25 ms, according to measurements with a BlackBox Toolkit v2. Observers were instructed to press the left-side “D” key (right-side “L” key) on a QWERTZ keyboard, which featured a sticker with the number 1 (2), if the first (second) stimulus appeared longer. Auditory feedback was delivered via loudspeakers.

#### Procedure

Each trial involved the first stimulus (*s* or *c*), the ISI (Experiments 1–2: 900 ms, Experiments 3–4: 200 ms), the second stimulus (*c* or *s*), a response-terminated interval, and an inter-trial interval (ITI; 900 ms). To encourage observers to perform at their best, this ITI was preceded by an error tone (1000 ms) followed by silence (1000 ms) if the response was incorrect and $s \ne c$. In Experiments 1 and 3, the LED lit up twice (filled intervals) but otherwise remained dark throughout the trial. Conversely, in Experiments 2 and 4, the LED was switched off twice (empty intervals) but was otherwise always illuminated.^4^

To rule out strategic shifts between $\langle sc \rangle$ and $\langle cs \rangle$, stimulus order varied randomly from trial to trial within each block, along with *c* (nine levels).^5^ Each of the 18 resulting trial types was repeated twice per block, yielding 36 trials presented in random order. Short and long stimulus durations were presented in two separate experimental parts, with 10 blocks of short stimulus durations followed by 10 blocks of long ones or vice versa (counterbalanced across observers). The resulting $36 \times 20 = 720$ experimental trials were preceded by two practice blocks (one for short and one for long stimulus durations) of 18 trials each (one per trial type). Observers could take a self-paced break between two blocks.

#### Analysis

Eight repeated-measures two-way analyses of variance (ANOVAs) examined the effects of *c* (nine levels) and stimulus order ($\langle sc \rangle$ vs. $\langle cs \rangle$) on the probability of judging *c* to be longer than *s*, separately for each combination of stimulus duration ($s = 80$ vs. 500 ms), ISI (200 vs. 900 ms), and stimulus type (filled vs. empty intervals). The *p* values were Greenhouse–Geisser corrected if a significant Mauchly test indicated a violation of sphericity. In these analyses, slope differences between $\langle sc \rangle$ and $\langle cs \rangle$ (i.e., Type B effects) are reflected in the interactions between *c* and stimulus order.

**Fig. 1:**
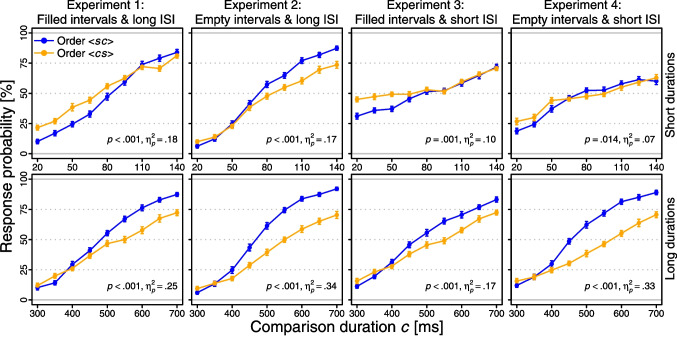
Group-level psychometric functions observed in Experiments 1–4. *Note*: *Error bars* reflect $\pm 1$ within-subjects standard error of the mean across observers (Cousineau, 2005; Morey, 2008). The *p* and $\eta _{p}^{2}$ values pertain to the *c* $\times$ stimulus order interaction in the respective two-way ANOVA

To quantify the Type B effect as a change in discrimination sensitivity, we also estimated the DLs using the non-parametric Spearman-Kärber method (Miller & Ulrich, 2001; Sternberg et al., 1982). This method allows a distribution-free SD estimation (where $\hat{\text {DL}} = z_{.75} \cdot \hat{\sigma } \approx 0.6745\,\hat{\sigma }$; see Bausenhart et al., 2018), requiring only assumptions about the *c* values at which the response probability reaches 0 and 1 (0 and 160 ms for short stimulus durations, and 200 and 800 ms for long ones).^6^ In these analyses, Type B effects correspond to the main effects of stimulus order on the DL estimates assessed in separate repeated-measures one-way ANOVAs.

A final analysis further investigated the determinants of the Type B effect. To enable more meaningful comparisons of Type B effects between short and long stimulus durations, the DL estimates were transformed to Weber fractions ($\text {WF} = \text {DL} / s$), following Hellström and Rammsayer (2004, 2015) and Hellström et al. (2020). The WF-based Type B effects ($\text {WF}_{\langle sc \rangle } - \text {WF}_{\langle cs \rangle }$) were then subjected to an overarching three-way mixed ANOVA, with the within-subjects factor stimulus duration and the between-subjects factors ISI and stimulus type.

### Results

Figure 1 presents the group-level psychometric functions observed in Experiments 1–4. The curves are steeper for $\langle sc \rangle$ than for $\langle cs \rangle$, consistently across all eight combinations of stimulus duration, ISI, and stimulus type. These negative Type B effects are reflected in significant interactions between *c* and stimulus order in all eight two-way ANOVAs performed on response probabilities (all *p*s < .015 and $\eta _{p}^{2}$s = .07–.34; see Fig. 1).

**Fig. 2:**
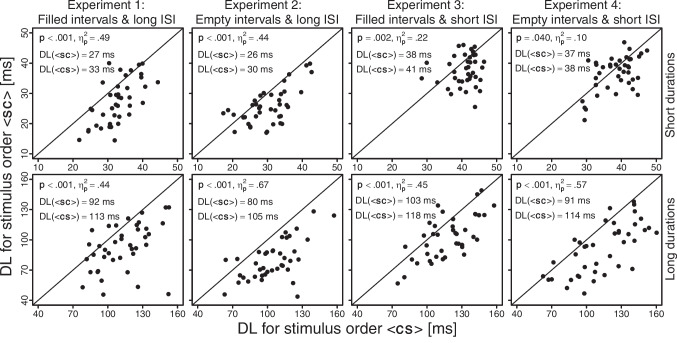
Individual DL estimates obtained from Experiments 1–4. *Note*: The *p* and $\eta _{p}^{2}$ values pertain to the effect of stimulus order on the DL estimates (see printed means across observers) in the respective one-way ANOVA

Figure 2 presents the individual DL estimates obtained from Experiments 1–4. In each experimental condition, most observers achieved a lower DL for $\langle sc \rangle$ than for $\langle cs \rangle$. All one-way ANOVAs revealed a significant effect of stimulus order on the DL estimates (all *p*s < .041 and $\eta _{p}^{2}$s = .10–.67; see Fig. 2), always in the direction of a lower DL for $\langle sc \rangle$ than for $\langle cs \rangle$. Thus, in line with the internal reference model, reliable negative Type B effects were consistently found across all experimental conditions and both analytical methods.

**Fig. 3:**
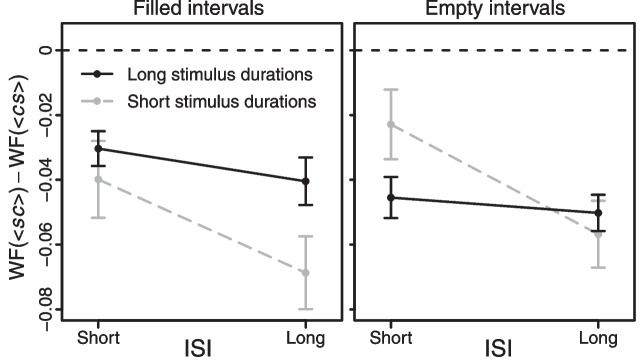
Group-level Type B effects based on the individual WFs obtained from Experiments 1–4. *Note*: *Error bars* reflect $\pm 1$ between-subjects standard error of the mean across observers

Finally, a three-way mixed ANOVA (stimulus duration $\times$ ISI $\times$ stimulus type) performed on WF-based Type B effects (see Fig. 3) revealed a less negative Type B effect when the ISI was short (significant main effect of ISI: $p =.010$, $\eta _{p}^{2}=.04$), especially for short stimulus durations (significant stimulus duration $\times$ ISI interaction: $p =.020$, $\eta _{p}^{2}=.03$). Unexpectedly, the WF-based Type B effect was also less negative when filled intervals were long, but conversely when empty intervals were short (significant stimulus duration $\times$ stimulus type interaction: $p =.009$, $\eta _{p}^{2}=.04$). No other effect reached significance (all *p*s > .285 and $\eta _{p}^{2}$s = .00–.01).

## Discussion

The present study systematically evaluated how visual duration discrimination sensitivity depends on the stimulus order of the standard *s* and the comparison *c* ($\langle sc \rangle$ vs. $\langle cs \rangle$). To this end, we orthogonally varied stimulus duration ($s = 80$ vs. 500 ms), ISI (200 vs. 900 ms), and stimulus type (filled vs. empty intervals) across four experiments. Two theories of comparative judgment make different predictions about the direction of this Type B effect ($\text {DL}_{\langle sc \rangle } - \text {DL}_{\langle cs \rangle }$): Whereas the internal reference model implies negative-to-null Type B effects, the more flexible sensation weighting model also allows for positive Type B effects. In line with the prediction of the internal reference model, all experimental conditions revealed a reliably better discrimination sensitivity for $\langle sc \rangle$ than for $\langle cs \rangle$; that is, the Type B effect was consistently negative. This is in line with internal reference updating in visual duration discrimination, and challenges the hypothesis that Type B effects become positive when employing short stimulus durations and/or a short ISI (Hellström et al., 2020).

The systematic absence of positive Type B effects in the present study extends the evidence obtained by Bausenhart et al. (2015) with the method of constant stimuli from auditory to visual duration discrimination. A minor empirical difference is that the combination of short stimulus durations and a short ISI eliminated the Type B effect in audition (Bausenhart et al., 2015), whereas this combination led to a reduced, yet still reliably negative Type B effect in vision (present study). While we did not observe consistent modulations of the Type B effect by stimulus type or duration, the Type B effect became less negative with a short ISI. Both the internal reference model and the sensation weighting model suggest explanations for this modulation.

In the internal reference model, a shorter ISI implies a shorter time span in which the first sensation magnitude can be integrated with the previous internal reference before the comparison with the second sensation magnitude. Thus, it seems plausible to assume that a shorter ISI decreases the extent of the reference-level integration (see Bausenhart et al., 2015; Dyjas et al., 2014) and/or increases the availability of the first sensation magnitude due to a less advanced decay of this perceptual representation (cf. Ellinghaus et al., 2019). In either case, the relative contribution of the stimulus history (*g* in Eq. 2) can be expected to decrease with a shorter ISI, which would lead to the observed ISI modulation of the negative Type B effect in the internal reference model.

In the sensation weighting model, the ISI modulation of the negative Type B effect can be explained by assuming that a shorter ISI increases the relative weighting of the first stimulus, although the second stimulus is still weighted more strongly than the first ($s_1 < s_2$ in Eq. 3). This account is broadly consistent with the hypothesis that shorter ISIs lead to less information loss about the first stimulus and more forward masking of the second stimulus (e.g., Hellström, 1979, 1985). However, it is inconsistent with $s_1 > s_2$ for short stimulus durations and/or short ISI, which was previously supported by fits of the sensation weighting model (Hellström, 1979; Hellström et al., 2020; Hellström & Rammsayer, 2004, 2015). Our findings instead suggest that even when successive stimuli are presented extremely rapidly, there is no need to allow $s_1 > s_2$ in the sensation weighting model to explain the effect of stimulus order on visual duration discrimination sensitivity.

Unlike the present study and Bausenhart et al. (2015), Hellström and Rammsayer (2004, 2015) and Hellström et al. (2020) did find positive Type B effects with short durations of visual and auditory stimuli (see Table 1). What could have caused this discrepancy? We suspect that the choice of adaptive-staircase procedures versus the method of constant stimuli played a role, since all previous observations of positive Type B effects listed in Table 1 were based on adaptive DL measurements. As described in the Introduction, pairing adaptive-staircase procedures with extremely small magnitudes may lead to biases in the estimated Type B effects. Our simulations indicate that the internal reference model can produce positive Type B effect estimates under such bias-prone conditions, casting some doubt on whether these previous findings were due to sensation weighting.

Although the method of constant stimuli is less prone to bias compared to adaptive-staircase procedures, the DL estimation is, of course, still challenging with extremely short durations. Future studies may therefore apply a small-*N* design by testing a few observers over many sessions (e.g., Kristofferson, 1980; Matthews & Grondin, 2012). This would likely further improve the capture of the psychometric functions (cf. Fig. 1), and the more precise individual DL estimates would enable more fine-grained tests of whether at least some observers produce reliable positive Type B effects (cf. Fig. 2). To further investigate the generality of the Type B effect, future studies may also use a binary “same”/“different” or a ternary “first”/“same”/“second” response format (e.g., Dyjas & Ulrich, 2014; García-Pérez & Alcalá-Quintana, 2010a; Hellström, 1979; Kelber & Ulrich, 2024; Rammsayer & Ulrich, 2001; Sternberg et al., in press; Ulrich, 1987). While a “same” choice option might invoke additional criteria and thereby alter discrimination sensitivity, its absence makes psychometric function slopes dependent on the guessing behavior in a hypothetical indifference interval (e.g., García-Pérez & Alcalá-Quintana, 2019; Kelber & Ulrich, 2025).

On a broader level, the idea that psychophysical judgments are informed by prior information is not only formalized in the sensation weighting model and the internal reference model, but also in various other models assuming static or dynamic memory representations (e.g., de Jong et al., 2021; Filippopoulos & Wearden, 2025; Glasauer & Shi, 2021; Jazayeri & Shadlen, 2010; Raviv et al., 2012; Wiener et al., 2014; for reviews, see Bausenhart et al., 2016; Shi et al., 2013; van Rijn, 2016). Of particular note are the models with dynamic updating rules (e.g., de Jong et al., 2021; Schumacher & Voss, 2023), as they explicitly capture the build-up of internal reference information and thus also lend themselves to the explanation of sequential trial-to-trial dependencies (de Jong et al., 2021; Dyjas et al., 2012; see also Bausenhart et al., 2014, for a reference updating explanation of Vierordt’s law) and overweighting of information from more recent trials (Raviv et al., 2012). Another possible avenue is to embed such models in an evidence accumulation framework (Schumacher & Voss, 2023; see also Patching et al., 2012), thereby linking a mechanistic trial-by-trial updating rule with a mechanistic model of the decision process within each trial. Given the variety of related models proposed to date, the present experimental results cannot provide conclusive evidence for the specific formulation of the internal reference model offered by Dyjas et al. (2012). What the present study demonstrates, however, is that the exclusion of positive Type B effects—as imposed by the internal reference model, for example—is consistently borne out in visual duration discrimination, even under optimal conditions for sensation weighting to produce positive Type B effects. This conclusion encourages theoretical developments situated between the restrictive difference model and the flexible sensation weighting model.

## Open practices:

Data, analysis and experimental scripts, and preregistrations are available at https://osf.io/4ca3r.

## Figures and Tables

**Fig. 4:**
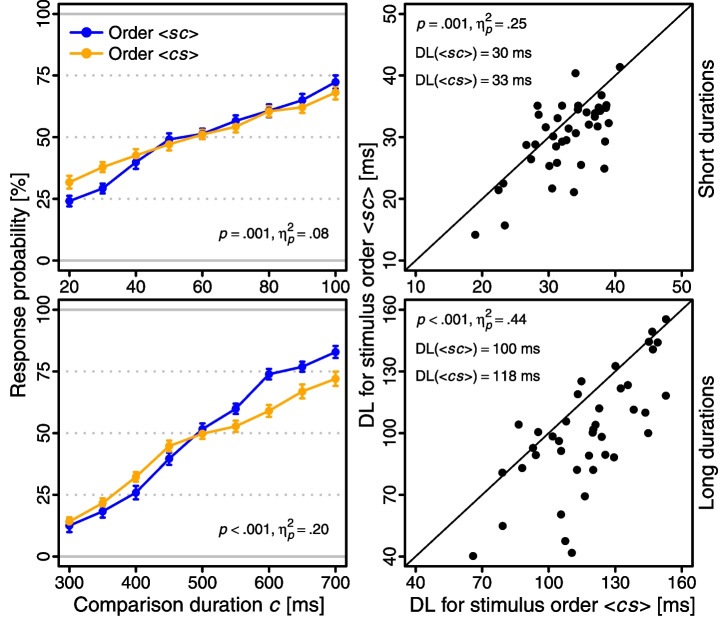
Group-level psychometric functions (*left*) and individual DL estimates (*right*) in the pilot experiment with filled intervals and long ISI and without accuracy feedback. *Note*: *Left*: *Error bars* reflect $\pm 1$ within-subjects standard error of the mean response probability across observers (Cousineau, 2005; Morey, 2008), and the *p* and $\eta _{p}^{2}$ values pertain to the *c* $\times$ stimulus order interaction in the respective two-way ANOVA. *Right*: The *p* and $\eta _{p}^{2}$ values pertain to the effect of stimulus order on the DL estimates (see printed means across observers) in the respective one-way ANOVA

## Data Availability

Data and experimental scripts are available at https://osf.io/4ca3r.

## Appendix

### A.1: Method

Forty observers (28 female, 40 right-handed, mean age [age range]: 23 [18–47] years, six replacements) participated in an experiment identical to Experiment 1 (filled intervals, short ISI) with two exceptions: For short durations, *s* was 60 ms and *c* ranged from 20 to 100 ms in steps of 10 ms. The observers did not receive accuracy feedback.

### A.2 Results

The group-level psychometric functions were again steeper for $\langle sc \rangle$ than for $\langle cs \rangle$ (see Fig. 4). These negative Type B effects were reliable across both stimulus durations and both analysis methods, as reflected in significant interaction effects of *c* and stimulus order on response probabilities (both *p*s < .002 and $\eta _{p}^{2}$s = .08–.20) and in significant main effects of stimulus order on DL estimates (both *p*s < .002 and $\eta _{p}^{2}$s = .25–.44).

## Footnotes

1 Without loss of generality, we tailored the following treatment of comparative judgment to the special case of duration discrimination investigated in the present experiments.

2 It has been suggested that extended difference models with an “indecision” (guessing) range can account for Type B effects (Alcalá-Quintana & García-Pérez, 2011; García-Pérez, 2014; García-Pérez & Alcalá-Quintana, 2010a, b, 2011, 2019). Accordingly, Type B effects result from a combination of several factors, such as a non-linear mapping between physical and perceived magnitudes, an asymmetric indifference interval around the perceptual midpoint, and biased guessing between the two choice options in typical binary-response (“first”/“second”) discrimination tasks. However, it remains unclear whether such extended difference models can convincingly explain why Type B effects have been so commonly observed across many stimuli and tasks (for an overview and a meta-analysis, see Ellinghaus et al., 2024), and why they almost always point in the same direction (as elaborated below). Furthermore, extended difference models are also challenged by sequential effects and Vierordt’s law, which the theoretical frameworks considered in the present study can account for (e.g., Bausenhart et al., 2014; de Jong et al., 2021; Dyjas et al., 2012; Raviv et al., 2012).

3 The internal reference model can account for Type A effects when allowing for response biases via $\gamma \ne 0$ ms (see Bausenhart et al., 2015, pp. 1039–1040).

4 Empty intervals were thus bounded by the offset and onset of sustained visual stimulation, rather than by brief light flashes. The latter, more conventional approach was not viable here: with the short stimulus durations and ISIs used in the present experiments, it produced an indistinct flicker, which made it unfeasible to determine the number of empty intervals reliably, let alone discriminate their durations.

5 Ulrich and Vorberg (2009) showed that the psychometric functions for $\langle sc \rangle$ and $\langle cs \rangle$ must average to 0.5 at $s=c$ in a proper 2AFC task. This constraint was satisfied by all observed pairs of psychometric functions in the present study, as reflected in 10 non-significant one-sample *t* tests (five experiments $\times$ two stimulus durations).

6 Although these assumptions affect the DL estimates when psychometric functions are not fully captured, sensitivity analyses with other reasonable start and end points always led to the same conclusion about the direction of the Type B effect. This conclusion also held true when DLs were estimated parametrically by fitting logistic functions.

## Code Availability

Analysis scripts are available at https://osf.io/4ca3r.
